# The role of CCR5 in HIV-associated neurocognitive disorders

**DOI:** 10.1016/j.heliyon.2022.e09950

**Published:** 2022-07-14

**Authors:** Cecile Riviere-Cazaux, Jessica Cornell, Yang Shen, Miou Zhou

**Affiliations:** a Graduate College of Biomedical Sciences, Western University of Health Sciences, Pomona, CA, USA; b Mayo Clinic Alix School of Medicine, Rochester, MN, USA; c Neurobiology, Psychiatry and Psychology Departments & Integrative Center for Learning and Memory, UCLA, Los Angeles, CA, USA

**Keywords:** CCR5, HIV-associated neurocognitive disorder (HAND), Maraviroc, Neuronal plasticity, Learning and memory, HIV gp120

## Abstract

While combination antiretroviral therapy (cART) has successfully increased the lifespan of individuals infected with HIV, a significant portion of this population remains affected by HIV-associated neurocognitive disorder (HAND). C-C chemokine receptor 5 (CCR5) has been well studied in immune response and as a co-receptor for HIV infection. HIV-infected (HIV^+^) patients experienced mild to significant amelioration of cognitive function when treated with different CCR5 antagonists, including maraviroc and cenicriviroc. Consistent with clinical results, *Ccr5* knockout or knockdown rescued cognitive deficits in HIV animal models, with mechanisms of reduced microgliosis and neuroinflammation. Pharmacologic inhibition of CCR5 directly improved cerebral and hippocampal neuronal plasticity and cognitive function. By summarizing the animal and human studies of CCR5 in HIV-associated cognitive deficits, this review aims to provide an overview of the mechanistic role of CCR5 in HAND pathophysiology. This review also discusses the addition of CCR5 antagonists, such as maraviroc, to cART for targeted prevention and treatment of cognitive impairments in patients infected with HIV.

## 1 Introduction

HAND is a broad syndrome of neurological deficits affecting individuals living with HIV, which may present with various stages of impaired cognitive, behavioral, and motor function, such as delayed speech output, inability to complete complex tasks, and deficits in learning and memory (Hong and Banks, 2015). HAND has been subclassified into three categories based on the degree of neurocognitive severity, including asymptomatic neurocognitive impairment (ANI) and mild neurocognitive disorder (MND), which account for most (over 90%) HAND cases, and HIV-associated dementia (HAD), which is the most cognitively deleterious diagnosis and has become less common with the advent of cART (Heaton et al., 2010). HIV^+^ individuals with ANI have impairment in two cognitive testing domains with a standard deviation (SD) one below the adjusted normative scores on standardized neuropsychological tests. However, in contrast to individuals with MND, they do not have impairment with their daily living (Antinori et al., 2007). HAD diagnoses were the most prevalent of HAND cases in the pre-cART era (Sacktor, 2018) and have since declined due to a successful suppression of viral load via cART treatment (Clifford and Ances, 2013; Deng et al., 1996; Heaton et al., 2010, 2011; Ru and Tang, 2017). Despite this improvement, neurocognitive impairment persists at a prevalence of 20-50% in HIV^+^ individuals (Cysique et al., 2014; De Francesco et al., 2016; Heaton et al., 2011; Robertson et al., 2007; Sacktor et al., 2016; Winston et al., 2013; Wright et al., 2015). A recent estimate suggests that 42.6% of individuals living with HIV continue to experience mild to moderate cognitive impairment associated with milder forms of HAND (Wang et al., 2020b). This indicates an unmet clinical need for neuroprotective treatments in HIV^+^ patients.

CCR5, a seven-membrane G protein-coupled receptor (GPCR), is highly expressed in microglia and is found to a lesser extent in neurons and astrocytes (Cartier et al., 2005; Fantuzzi et al., 2019; Tran et al., 2007; Westmoreland et al., 2002). CCR5 is enriched in the hippocampal CA1 (Torres-Muñoz et al., 2004), a brain region known to be critical for the acquisition and consolidation of episodic and spatial memories, the latter of which are crucial to successful navigation. In several recent studies exploring the role of CCR5 in normal learning and memory, CCR5 has been discovered to function as a potent suppressor for hippocampal and cortical neuronal plasticity, in addition to hippocampus-dependent learning and memory (Frank et al., 2018; Joy et al., 2019; Necula et al., 2020; Zhou et al., 2016). CCR5 is a critical co-receptor for HIV infection via gp120 binding, especially for HIV infection in the central nervous system (CNS) (Deng et al., 1996; Ellis et al., 2007). As such, murine preclinical and human clinical studies have been carried out to elucidate CCR5's role in HIV-associated cognitive dysfunction and to determine if CCR5 inhibition may ameliorate learning and memory deficits in HIV animal models and HIV^+^ patients. This review will introduce the current evidence implicating CCR5 in HAND and the potential neuroprotective role of CCR5 antagonists when combined with the cART regimen.

## 2 Mechanistic studies of the role of CCR5 in HAND with animal models

**Figure 1:**
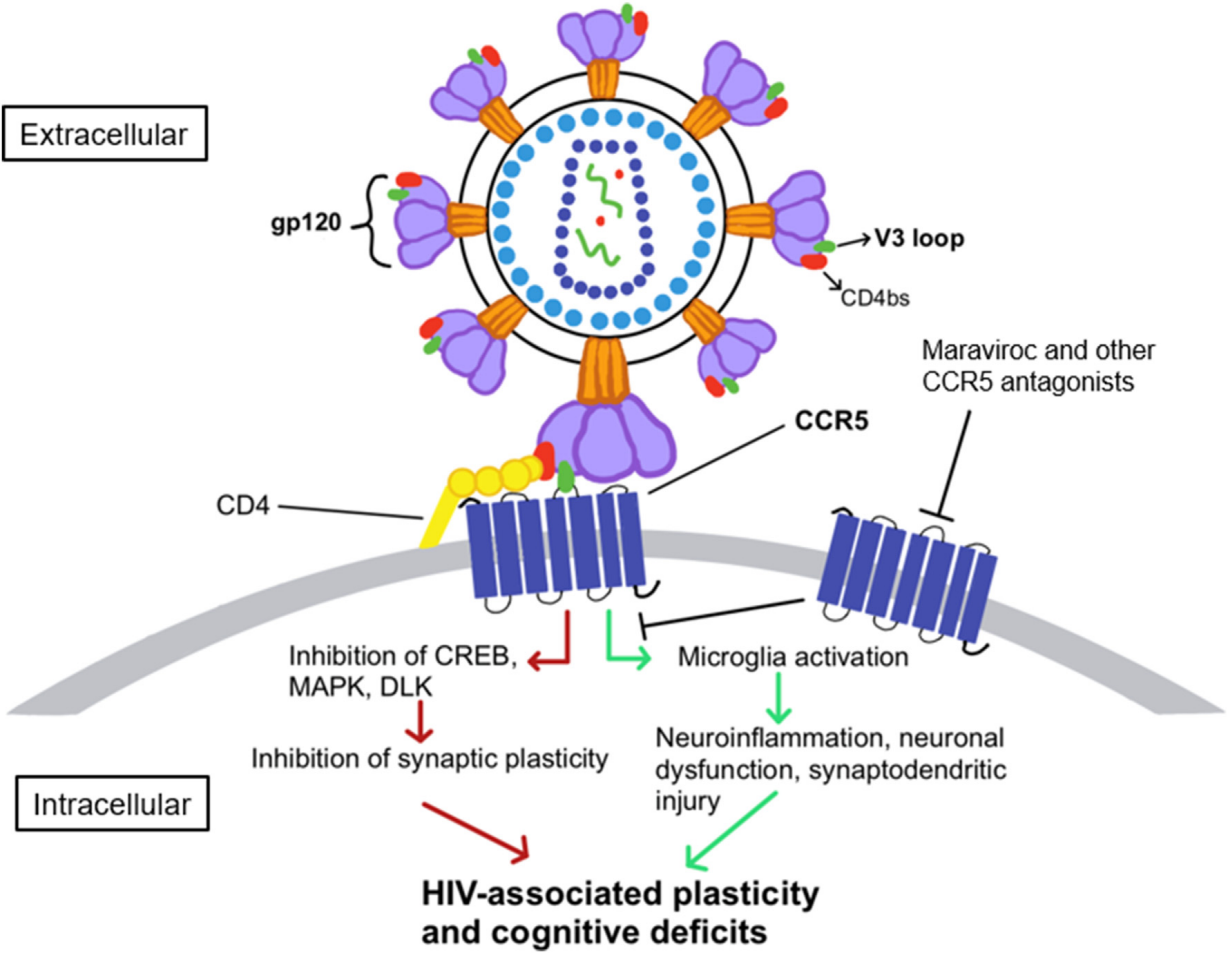
CCR5 and HAND. Binding of HIV-1 via its gp120 V3 domain to CCR5 leads to activation of this chemokine receptor. This triggers two different signaling pathways that may lead to the cognitive deficits in HAND. An acute, direct pathway (red arrows) inhibits CREB, MAPK, and dual leucine zipper kinase (DLK), subsequently inhibiting synaptic plasticity. A chronic, indirect pathway (green arrows) activates microglia, causing neuroinflammation, neuronal dysfunction, and synaptodendritic injury. Both pathways result in HIV-associated plasticity and cognitive function deficits. Maraviroc and other CCR5 antagonists block gp120 binding to CCR5, ameliorating these deficits.

CCR5 is reported to act as a suppressor for synaptic plasticity and learning and memory (Frank et al., 2018; Zhou et al., 2016) (Table 1). In a reverse genetic screening for novel genes regulating learning and memory, *Ccr5* knockout mice demonstrated enhanced memory when tested 24 h or 2 weeks after contextual fear conditioning (Zhou et al., 2016). Additionally, *Ccr5* knockout mice had enhanced long-term potentiation (LTP) both in the barrel cortex and hippocampus. LTP is believed to be an underlying cellular mechanism for learning and memory. Consistent with the enhanced LTP, *Ccr5* knockout mice also showed enhancement in experience-dependent cortical plasticity, as well as in hippocampus-dependent learning tasks, including Morris water maze, social recognition, and contextual fear conditioning (Frank et al., 2018; Zhou et al., 2016). Furthermore, both *Ccr5* knockdown and treatment with maraviroc enhanced dendritic spine formation and axon regeneration after brain injury. As a result, CCR5 inhibition improved motor and cognitive recovery in a mouse model of stroke (Joy et al., 2019). These results suggested that besides its well-known role in immune response and HIV infection, CCR5 also functions as a suppressor for neuronal plasticity and learning and memory (Figure 1). Consistent with these findings, CCR5 overexpression in excitatory neurons caused learning and memory deficits both in water maze and fear conditioning (Zhou et al., 2016).

**Table 1:** Studies of the role of CCR5 in HAND or neuronal plasticity and excitability with animal models.

Methods	Main Findings	Author & Date
Mice: Transgenic mice expressing HIV gp120 in the brain (with or without *Ccr5* KO plus *Lcn2* KO)	Knockout of LCN2 in HIVgp120tg mice abrogates memory impairment and ameliorates neuronal damage. Genetic ablation of *Ccr5* in LCN2-deficient HIVgp120tg mice restores neuropathology	Ojeda-Juárez et al., 2020
Mice: V3 peptide injection to the dorsal hippocampus or barrel cortex of mice with *Ccr5* knockout or *Ccr5* knockdown	*Ccr5* knockout rescued LTP deficits caused by gp120 V3 peptide both in hippocampus and cortex. Both *Ccr5* knockout and knockdown ameliorated V3 peptide induced memory deficits	Zhou et al., 2016
Mice: Transgenic mice expressing gp120, and double transgenic mice expressing gp120 and *Ccr5* knockout	*Ccr5* knockout prevented microglial activation and neuronal damage in HIVgp120tg mice. LCN2 neurotoxicity is CCR5 dependent, and *Ccr5* knockout rescued learning and memory deficits in HIVgp120tg mice	Maung et al., 2014
Rats: Cultured hippocampal neurons from rat embryos with acute gp120 treatment	Modifications in Kv2.1 and neuronal excitability after gp120 treatment were dependent on the activation of CCR5 and CXCR4. Blockade of Kv2.1 led to significant enhancement of neuronal death upon gp120 treatment	Shepherd et al., 2013

∗LCN2: Protein lipocalin-2, LTP: long-term potentiation.

The role of CCR5 and its ligands in HIV-induced cognitive deficits has been studied in HIV mouse models. Increased expression of CCR5 and neuroinflammation was observed in the brain of Tg26 mice, a model for HAND (Bryant et al., 2021). The HIV gp120 V3 loop peptide contains the gp120 domain that can bind to and activate CCR5 (Shen et al., 2000). Since CCR5 is a suppressor for plasticity and learning and memory, it is possible that acute activation of CCR5 by HIV coat proteins could contribute to deficits in neuroplasticity and thereby learning and memory, potentially as seen in the development of HAND after HIV infection. In wild-type (WT) mice, V3 peptide treatment completely abolished the induction of LTP in response to a spike-timing dependent LTP-induction protocol and produced an average effect of long-term depression (LTD) (Zhou et al., 2016). In contrast to WT mice, *Ccr5* knockout rescued the V3 peptide-induced LTP impairment and eliminated the production of LTD. Consistent with the rescue effect of neuronal plasticity, both *Ccr5* knockout and knockdown ameliorated the learning and memory deficits caused by V3 peptide treatment in the dorsal hippocampus. Since the V3 peptide was injected into the hippocampus 30 min before learning, these results indicate that in addition to chronic neuroinflammation and neuronal damages, CCR5 may also be involved in the plasticity and cognitive deficits caused by acute V3 treatment. Since gp120 treatment activated the Kv2.1 channel via rapid dephosphorylation and modulated neuronal excitability in a CCR5 and CXCR4-dependent manner (Shepherd et al., 2013), CCR5 may regulate neuronal plasticity by inhibiting neuronal excitability after gp120 or V3 treatment (Table 1). Taken together, these results suggest that after activation by the HIV protein gp120, CCR5 may inhibit neuronal excitability and plasticity and contribute to the cognitive deficits associated with HAND.

Another mechanism by which CCR5 may cause HIV-associated cognitive impairments is increased microglia activation and neuroinflammation (Figure 1). Gp120 binding to CCR5 may cause microglial activation, resulting in an increased release of reactive oxygen species, viral proteins, proinflammatory chemokines, and cytokines contributing to neuronal apoptosis (Acquas et al., 2004; Kaul et al., 2007; Kim et al., 2018; Saylor et al., 2016). In a gp120-transgenic murine model of HIV (HIVgp120tg mice), knockout of *Ccr5* subsequently reduced microglia activation and prevented the neuronal damages caused by CCR5-dependent protein lipocalin-2 (LCN2) neurotoxicity (Maung et al., 2014) (Table 1). As a result, *Ccr5* knockout rescued the spatial memory deficits caused by gp120 overexpression in the Barnes maze test. In genome-wide gene expression analysis, LCN2 was found to be one of the most significantly upregulated factors in HIVgp120tg mice, and the combination of maraviroc and LCN2 protected neurons from gp120-induced toxicity (Maung et al., 2014). This suggested a neuroprotective role of LCN2 in combination with CCR5 inhibition in HAND. Intriguingly and somewhat paradoxically, the same group later found that LCN2 knockout rescued memory deficits in HIVgp120tg mice, *Ccr5* knockout restored neuronal damage and increased gliosis in LCN2-deficient HIVgp120tg mice (Ojeda-Juárez et al., 2020). While CCR5 mediates HIV-1 infection of macrophages and microglia, similar to CCR5 antagonists (e.g., maraviroc), CCR5 ligands (e.g., CCL4 and CCL5) can also act as inhibitors of CCR5-preferring viruses and thus reduce CCR5-mediated HIV-1 infection (Maung et al., 2014; Ojeda-Juárez et al., 2020; Tyner et al., 2005). These studies suggest that depending on CCR5 ligand concentration and other signaling pathways (e.g., LCN2), CCR5 and its ligands may have dose-dependent roles in neuronal damage and cognitive deficits caused by HIV infection.

A recent study suggests that synaptodendritic damage may underly the cognitive deficits caused by HIV infection of the CNS (Avdoshina et al., 2020; Smith et al., 2021). HIV gp120 bound to either HIV co-receptor CCR5 or CXCR4 induced the formation of aberrant, rod-shaped cofilin-actin inclusions (rods) in cultured mouse hippocampal neurons. The signaling pathways involved, including active NADPH oxidase, superoxide (O_2_^-^) formation, and expression of cellular prion protein (PrP^C^), are common to other neurodegenerative stimuli such as oligomeric, soluble amyloid-β (Aβ). Cofilin-actin rods are observed in neurons exposed to Aβ oligomers and can contribute to synaptotoxicity and cognitive deficits in Alzheimer's Disease (Rush et al., 2018; Wang et al., 2020a). Maraviroc both reduced cofilin-actin rod formation in neurons exposed to R5-tropic gp120 and blocked rod induction by soluble Aβ (Smith et al., 2021), suggesting that CCR5 may be involved in gp120-induced synaptodendritic damage. Another study found that CCR5 was involved in gp120-induced down-regulation of phosphorylated NMDA receptor subunit 1 (NR1) in cortical neurons (Ru and Tang, 2016). NMDA receptors are essential for synaptic plasticity during learning and memory (Herron et al., 1986; Huang et al., 1992; Lynch and Baudry, 1987). Overactivation of NMDA receptors and dysfunctional glutamate metabolism can contribute to dendritic spine loss and HAND (Raybuck et al., 2017; Zhou et al., 2020). Maraviroc inhibited the effect of gp120 on NR1 dephosphorylation, suggesting that CCR5 activation was involved in synaptopathogenesis caused by HIV-1 infection.

It must be noted that in early HIV studies, the interaction of gp120 and CCR5 might cause cognitive impairments by causing neuronal apoptosis (Mocchetti et al., 2013; Saylor et al., 2016). With cART, neuronal loss may no longer be the main contributor to HAND (Kelschenbach et al., 2019; Necula et al., 2021). Instead, neuronal dysfunction and synaptodendritic injury after microglia activation may be a more plausible source of cognitive impairment (Ellis et al., 2007; Kelschenbach et al., 2019). Altogether, these CCR5-related structural and physiological changes may underlie HAND in the era of cART. Additionally, while HIV most often utilizes CCR5 in its pathogenicity, this virus is well-known for the diversity present in its envelope glycoproteins which can bind and use other chemokine receptors (such as CXCR4) to infect cells (Maeda et al., 2020), and the development of dual-mixed viral tropism (CCR5 and CXCR4) may be associated with HAND early on during the infectious process (Morris et al., 2013). One study has demonstrated that CXCR4 may play a role in neuronal dysfunction during HAND by inducing a G-inhibitory protein-linked decrease in cyclic AMP and increasing inositol 1,4,5-triphosphate (IP_3_) and intracellular calcium (Zheng et al., 1999). Another study has demonstrated that gp120 binding to either CCR5 or CXCR4 induced rod formation via an NADPH-oxidase dependent mechanism for the formation of superoxide and cellular prion protein (Smith et al., 2021). Festa et al. demonstrated that the chemokine CXCL12 increased cognitive performance and synaptodendritic health, specifically via a CXCR4-dependent stimulation of the Rac1/PAK actin polymerization pathway which increased spine density and flexible behavior (Festa et al., 2020). These studies suggest that similar to CCR5, CXCR4 is also involved in altered synaptic plasticity and cognitive performance in HAND. Since CCR5-using HIV-1 variants predominate in most of infection, while CXCR4-using HIV-1 variants (including variants using both CCR5 and CXCR4 or CXCR4 alone) emerge at the relatively late-stage infection (Maeda et al., 2020), the different roles of CCR5 and CXCR4 in the development and cognitive phenotypic expression of HAND may need to be evaluated with future studies.

## 3 Clinical studies of the role of CCR5 in HAND

The vast majority of HIV strains in the brain are CCR5 tropic (Carroll and Brew, 2017; González-Scarano and Martín-García, 2005). When endogenous CCR5 ligands were measured in cerebral spinal fluid (CSF) samples from HIV-infected patients, CCL3 (MIP-1α), CCL4 (MIP-1β) and CCL5 (RANTES) were found in higher concentrations within the CSF of patients with dementia, and the CSF chemokine levels did not differ whether patients used antiretroviral agents or not (Letendre et al., 1999). CSF samples with undetectable CCL3, CCL4 or CCL5 levels were almost entirely from nondemented patients, and detectable CSF CCL3 and CCL4 were both associated with dementia. However, the association between dementia and CSF CCL3 levels were nonlinear. Patients with either undetectable or high detectable CCL3 levels were more likely to be nondemented. In contract, patients with dementia were more likely to have low to midrange CCL3 levels. These results suggest that when CCL3 levels are low CCR5 has a deleterious effect on cognition, while with high levels of CCL3 or other CCR5 ligands, those ligands may block the entry of HIV-1 into cells and therefore provide neuroprotection (Cocchi et al., 1996). When a CCR5 antagonist called D-Ala1-peptide T-amide (DAPTA) was administered to HIV-1 seropositive participants with cognitive impairment, subgroups with greater cognitive impairment at baseline showed significant improvement, although no cognitive effect was observed in the overall cohort (Goodkin et al., 2006) (Table 2). Although CCR5 ligand levels were not measured in this study, the effect of CCR5 antagonist in different subgroups (mild, moderate, versus severe cognitive impairment) in the overall cohort could depend on the levels of CCR5 endogenous ligands in the CSF.

**Table 2:** Clinical studies on the role of CCR5 in HAND.

Methods	Main Findings	Author & Date
Measured MAP2 concentrations in human CSF, and immune-reactivity in rat cortical neurons exposed to gp120	Persons living with HIV (PLH) who had HAND had greater CSF MAP2 concentrations than cognitive normal PLH. The neurotoxic effect of HIV was blocked by a CCR5 antagonist	Avdoshina et al., 2020
HIV infected individuals with below-normal cognitive performance were assessed after 24 weeks of treatment	Patients with cenicriviroc treatment showed improved neuropsychological test performance	D'Antoni et al., 2018
Addition of maraviroc to DRV/r monotherapy for 24 weeks and neurocognitive function was measured	HIV-infected patients with maraviroc and DRV/r monotherapy had improvement in executive function but with no global neurocognitive effect	Barber et al., 2018
Intensification of cART with maraviroc. Performance was assessed before or at 6 and 12 months after the treatment	Patients treated with maraviroc and cART showed improved global neurocognitive performance	Gates et al., 2016
Intensification of cART with maraviroc. Performance was assessed before or at 24 weeks after the treatment	Patients who entered the study with evidence of mild to moderate cognitive impairment showed improvement in neuropsychological performance	Ndhlovu et al., 2014
DAPTA (CCR5 antagonist) versus placebo prior to combination antiretroviral therapy with HIV-1 seropositive participants having cognitive impairment	No overall cognitive effect was observed, but subgroups with greater cognitive impairment at baseline showed significant improvement	Goodkin et al. (2006)

∗cART: combined antiretroviral therapy, CSF: cerebrospinal fluid, DAPTA: D-Ala1-peptide T-amide, DRV/r: darunvair/ritonavir, HAND: HIV-associated neurocognitive disorders, LTP: long-term potentiation, MAP2: microtubule-associated protein 2.

Recently, multiple studies have studied the effects of an FDA-approved CCR5 antagonist, maraviroc, on neurocognitive performance in virally-suppressed HAND patients. In a prospective, observer-blinded, open-label phase IV clinical trial (NCT01449006), participants were allocated to either continue with their current cART regimen (control arm) or to receive cART plus maraviroc intensification (maraviroc arm). The participants completed a five-domain neuropsychological battery before or at 6 and 12 months after the treatment. CogState, a computerized battery sensitive to HAND, was used in addition to standardized paper-and-pencil neuropsychological tests. The battery assessed five domains of HAND: speed of informational processing, attention/working memory, motor coordination, verbal learning, and verbal memory. Compared to the control arm, improved global neurocognitive performance was observed in the maraviroc arm at both the 6- and 12-month testing points (Gates et al., 2016).

In a single-arm, open-labeled clinical trial, cART was intensified with maraviroc to determine the effect of CCR5 antagonism on monocyte activation, inflammation, and cognition (Ndhlovu et al., 2014) (Table 2). Subjects in this study were administered maraviroc for 24 weeks in addition to their baseline cART regimen. Increased CD16 expressing monocytes activation and soluble CD163 (sCD163) have been shown to be associated with neurocognitive impairment upon HIV infection (Burdo et al., 2013; Pulliam et al., 2004). When monocyte activation was measured at week 24, a significant decrease in intermediate (CD14^++^CD16^+^) monocyte subset and nonclassical (CD14^+/low^CD16^+^) monocyte subset was observed in 11 of the 12 study participants. A decline in sCD163 levels was also observed, indicating that maraviroc led to declines in proinflammatory monocyte activation (Ndhlovu et al., 2014). Participant's cognition was tested at entry and at the 24th week using a neuropsychological battery assessing attention and concentration, learning and memory, psychomotor speed, executive functioning, language, and gross motor functions. No significant changes were observed in global composite scores for the neuropsychological battery, although some improvement in executive function was noted. However, when patients were stratified based on cognition level at the beginning of the study, six patients with mild-to-moderate baseline cognitive impairment showed neuropsychological improvement in global functioning and cognitive domains including global functioning, learning and memory, and executive function (Ndhlovu et al., 2014).

Besides CCR5 antagonists DAPTA and maraviroc, a dual CCR2 and CCR5 antagonist cenicriviroc was tested for HIV-infected individuals with below-normal cognitive performance at baseline as determined by neuropsychological battery performance (DʼAntoni et al., 2018). Individuals received different amounts of cenicriviroc (50 mg, 200 mg, or 800 mg) depending on their current cART regimen. Neuropsychological testing was conducted at entry and after 24 weeks to calculate both global and domain-specific neuropsychological Z-scores (NPZ). The global, cognitive domains of attention and working memory NPZs all significantly improved over 24 weeks. Additionally, plasma levels of sCD163, sCD14, and neopterin decreased, suggesting decreased monocyte/macrophage activation. All together, these findings suggest that CCR5 antagonist may be associated with both favorable changes in monocyte activation and improved neuropsychological performance in patients with baseline cognitive impairment. Further studies are needed to determine the role of proinflammatory monocyte and immune activation in cognitive function for HIV^+^ individuals.

In another open-label, phase IV clinical trial, 150 mg of daily maraviroc was added to the patients’ drug regimen after 12 weeks of darunvair/ritonavir monotherapy (DRV/r) (Barber et al., 2018) (Table 2). After 36 weeks (12 weeks control followed by 24 weeks total MVC + DVR/r), patients showed some improvement in executive function based on age-adjustment compared to control, although there were no changes in overall neurocognitive outcome or in CSF inflammatory markers. Altogether, while some studies provide preliminary supportive evidence for maraviroc plus cART in neurocognition for patients with HAND, this positive effect is not seen across all studies or measures of cognitive performance. As such, elucidation of the role of CCR5 across cognitive domains warrants further investigation to select and determine patients in which maraviroc intensification may be most beneficial.

## 4 Conclusion

CCR5 has been widely studied in HIV infection since its discovery as a co-receptor for HIV infection of target cells (Deng et al., 1996) and several CCR5 antagonists have been designed to block HIV entry into host cells. Despite this, the role of CCR5 in HIV-associated cognitive impairment is less understood. Despite mixed results warranting further investigation, some clinical studies have reported that CCR5 antagonists such as DAPTA, cenicriviroc, and maraviroc may enhance neuropsychological test performance in virally suppressed HIV^+^ patients (Barber et al., 2018; DʼAntoni et al., 2018; Gates et al., 2016; Goodkin et al., 2006). Most preclinical studies to date on CCR5 in HAND have focused on the gp120-induced neuroinflammation and the resulting neurodegeneration and neuronal dysfunction (Ahmad et al., 2019; Gonek et al., 2018; Gu et al., 2016; Tang et al., 2015). In addition to the microglia activation and inflammation mechanism, some recent studies have shown that CCR5 activation can cause neuronal CREB and MAPK inactivation, in addition to impaired axonal regrowth after neuronal damage. Additionally, direct interaction between the gp120 V3 domain and CCR5 could compromise synaptic plasticity directly and therefore suppress memory without neuroinflammation (Figure 1) (Joy et al., 2019; Zhou et al., 2016). CCR5 and its FDA-approved antagonist, maraviroc, have received high attention as a candidate for pharmacological intervention for HIV infection (Martin-Blondel et al., 2016). We expect that future studies examining the transient or long-term blockade of CCR5, especially in combination with cART regimen, will have particular translational significance for the targeted treatment and prevention of HAND in patients living with HIV.

## Declarations

### Author contribution statement

All authors listed have significantly contributed to the development and the writing of this article.

### Funding statement

This research did not receive any specific grant from funding agencies in the public, commercial, or not-for-profit sectors.

### Data availability statement

No data was used for the research described in the article.

### Declaration of interest’s statement

The authors declare no conflict of interest.

### Additional information

No additional information is available for this paper.

## References

[bib1] Acquas E., Bachis A., Nosheny R.L., Cernak I., Mocchetti I. (2004). Human immunodeficiency virus type 1 protein gp120 causes neuronal cell death in the rat brain by activating caspases. Neurotox. Res..

[bib2] Ahmad S.F., Ansari M.A., Nadeem A., Bakheet S.A., Alotaibi M.R., Alasmari A.F., Alshammari M.A., Al-Mazroua H.A., Attia S.M. (2019). DAPTA, a C-C chemokine receptor 5 (CCR5) antagonist attenuates immune aberrations by downregulating Th9/Th17 immune responses in BTBR T(+) Itpr3tf/J mice. Eur. J. Pharmacol..

[bib3] Antinori A., Arendt G., Becker J.T., Brew B.J., Byrd D.A., Cherner M., Clifford D.B., Cinque P., Epstein L.G., Goodkin K. (2007). Updated research nosology for HIV-associated neurocognitive disorders. Neurology.

[bib4] Avdoshina V., Mahoney M., Gilmore S.F., Wenzel E.D., Anderson A., Letendre S.L., Imamichi T., Fischer N.O., Mocchetti I. (2020). HIV influences microtubule associated protein-2: potential marker of HIV-associated neurocognitive disorders. Aids.

[bib5] Barber T.J., Imaz A., Boffito M., Niubó J., Pozniak A., Fortuny R., Alonso J., Davies N., Mandalia S., Podzamczer D. (2018). CSF inflammatory markers and neurocognitive function after addition of maraviroc to monotherapy darunavir/ritonavir in stable HIV patients: the CINAMMON study. J. Neurovirol..

[bib6] Bryant J., Andhavarapu S., Bever C., Guda P., Katuri A., Gupta U., Arvas M., Asemu G., Heredia A., Gerzanich V. (2021). 7,8-Dihydroxyflavone improves neuropathological changes in the brain of Tg26 mice, a model for HIV-associated neurocognitive disorder. Sci. Rep..

[bib7] Burdo T.H., Lackner A., Williams K.C. (2013). Monocyte/macrophages and their role in HIV neuropathogenesis. Immunol. Rev..

[bib8] Carroll A., Brew B. (2017). HIV-associated neurocognitive disorders: recent advances in pathogenesis, biomarkers, and treatment. F1000Res.

[bib9] Cartier L., Hartley O., Dubois-Dauphin M., Krause K.H. (2005). Chemokine receptors in the central nervous system: role in brain inflammation and neurodegenerative diseases. Brain Res. Brain Res. Rev..

[bib10] Clifford D.B., Ances B.M. (2013). HIV-associated neurocognitive disorder. Lancet Infect. Dis..

[bib11] Cocchi F., DeVico A.L., Garzino-Demo A., Cara A., Gallo R.C., Lusso P. (1996). The V3 domain of the HIV-1 gp120 envelope glycoprotein is critical for chemokine-mediated blockade of infection. Nat. Med..

[bib12] Cysique L.A., Heaton R.K., Kamminga J., Lane T., Gates T.M., Moore D.M., Hubner E., Carr A., Brew B.J. (2014). HIV-associated neurocognitive disorder in Australia: a case of a high-functioning and optimally treated cohort and implications for international neuroHIV research. J. Neurovirol..

[bib13] DʼAntoni M.L., Paul R.H., Mitchell B.I., Kohorn L., Fischer L., Lefebvre E., Seyedkazemi S., Nakamoto B.K., Walker M., Kallianpur K.J. (2018). Improved cognitive performance and reduced monocyte activation in virally suppressed chronic HIV after dual CCR2 and CCR5 antagonism. J. Acquir. Immune Defic. Syndr..

[bib14] De Francesco D., Underwood J., Post F.A., Vera J.H., Williams I., Boffito M., Sachikonye M., Anderson J., Mallon P.W., Winston A. (2016). Defining cognitive impairment in people-living-with-HIV: the POPPY study. BMC Infect. Dis..

[bib15] Deng H., Liu R., Ellmeier W., Choe S., Unutmaz D., Burkhart M., Di Marzio P., Marmon S., Sutton R.E., Hill C.M. (1996). Identification of a major co-receptor for primary isolates of HIV-1. Nature.

[bib16] Ellis R., Langford D., Masliah E. (2007). HIV and antiretroviral therapy in the brain: neuronal injury and repair. Nat. Rev. Neurosci..

[bib17] Fantuzzi L., Tagliamonte M., Gauzzi M.C., Lopalco L. (2019). Dual CCR5/CCR2 targeting: opportunities for the cure of complex disorders. Cell. Mol. Life Sci..

[bib18] Festa L.K., Irollo E., Platt B.J., Tian Y., Floresco S., Meucci O. (2020). CXCL12-induced rescue of cortical dendritic spines and cognitive flexibility. Elife.

[bib19] Frank A.C., Huang S., Zhou M., Gdalyahu A., Kastellakis G., Silva T.K., Lu E., Wen X., Poirazi P., Trachtenberg J.T. (2018). Hotspots of dendritic spine turnover facilitate clustered spine addition and learning and memory. Nat. Commun..

[bib20] Gates T.M., Cysique L.A., Siefried K.J., Chaganti J., Moffat K.J., Brew B.J. (2016). Maraviroc-intensified combined antiretroviral therapy improves cognition in virally suppressed HIV-associated neurocognitive disorder. Aids.

[bib21] Gonek M., McLane V.D., Stevens D.L., Lippold K., Akbarali H.I., Knapp P.E., Dewey W.L., Hauser K.F., Paris J.J. (2018). CCR5 mediates HIV-1 Tat-induced neuroinflammation and influences morphine tolerance, dependence, and reward. Brain Behav. Immun..

[bib22] González-Scarano F., Martín-García J. (2005). The neuropathogenesis of AIDS. Nat. Rev. Immunol..

[bib23] Goodkin K., Vitiello B., Lyman W.D., Asthana D., Atkinson J.H., Heseltine P.N., Molina R., Zheng W., Khamis I., Wilkie F.L. (2006). Cerebrospinal and peripheral human immunodeficiency virus type 1 load in a multisite, randomized, double-blind, placebo-controlled trial of D-Ala1-peptide T-amide for HIV-1-associated cognitive-motor impairment. J. Neurovirol..

[bib24] Gu S.M., Park M.H., Yun H.M., Han S.B., Oh K.W., Son D.J., Yun J.S., Hong J.T. (2016). CCR5 knockout suppresses experimental autoimmune encephalomyelitis in C57BL/6 mice. Oncotarget.

[bib25] Heaton R.K., Clifford D.B., Franklin D.R., Woods S.P., Ake C., Vaida F., Ellis R.J., Letendre S.L., Marcotte T.D., Atkinson J.H. (2010). HIV-associated neurocognitive disorders persist in the era of potent antiretroviral therapy: CHARTER Study. Neurology.

[bib26] Heaton R.K., Franklin D.R., Ellis R.J., McCutchan J.A., Letendre S.L., Leblanc S., Corkran S.H., Duarte N.A., Clifford D.B., Woods S.P. (2011). HIV-associated neurocognitive disorders before and during the era of combination antiretroviral therapy: differences in rates, nature, and predictors. J. Neurovirol..

[bib27] Herron C.E., Lester R.A., Coan E.J., Collingridge G.L. (1986). Frequency-dependent involvement of NMDA receptors in the hippocampus: a novel synaptic mechanism. Nature.

[bib28] Hong S., Banks W.A. (2015). Role of the immune system in HIV-associated neuroinflammation and neurocognitive implications. Brain Behav. Immun..

[bib29] Huang Y.Y., Colino A., Selig D.K., Malenka R.C. (1992). The influence of prior synaptic activity on the induction of long-term potentiation. Science.

[bib30] Joy M.T., Ben Assayag E., Shabashov-Stone D., Liraz-Zaltsman S., Mazzitelli J., Arenas M., Abduljawad N., Kliper E., Korczyn A.D., Thareja N.S. (2019). CCR5 is a therapeutic target for recovery after stroke and traumatic brain injury. Cell.

[bib31] Kaul M., Ma Q., Medders K.E., Desai M.K., Lipton S.A. (2007). HIV-1 coreceptors CCR5 and CXCR4 both mediate neuronal cell death but CCR5 paradoxically can also contribute to protection. Cell Death Differ..

[bib32] Kelschenbach J., He H., Kim B.H., Borjabad A., Gu C.J., Chao W., Do M., Sharer L.R., Zhang H., Arancio O. (2019). Efficient expression of HIV in immunocompetent mouse brain reveals a novel nonneurotoxic viral function in hippocampal synaptodendritic injury and memory impairment. mBio.

[bib33] Kim S., Hahn Y.K., Podhaizer E.M., McLane V.D., Zou S., Hauser K.F., Knapp P.E. (2018). A central role for glial CCR5 in directing the neuropathological interactions of HIV-1 Tat and opiates. J. Neuroinflammation.

[bib34] Letendre S.L., Lanier E.R., McCutchan J.A. (1999). Cerebrospinal fluid beta chemokine concentrations in neurocognitively impaired individuals infected with human immunodeficiency virus type 1. J. Infect. Dis..

[bib35] Lynch G., Baudry M. (1987). Brain spectrin, calpain and long-term changes in synaptic efficacy. Brain Res. Bull..

[bib36] Maeda Y., Takemura T., Chikata T., Kuwata T., Terasawa H., Fujimoto R., Kuse N., Akahoshi T., Murakoshi H., Tran G.V. (2020). Existence of replication-competent minor variants with different coreceptor usage in plasma from HIV-1-Infected individuals. J. Virol..

[bib37] Martin-Blondel G., Brassat D., Bauer J., Lassmann H., Liblau R.S. (2016). CCR5 blockade for neuroinflammatory diseases--beyond control of HIV. Nat. Rev. Neurol..

[bib38] Maung R., Hoefer M.M., Sanchez A.B., Sejbuk N.E., Medders K.E., Desai M.K., Catalan I.C., Dowling C.C., de Rozieres C.M., Garden G.A. (2014). CCR5 knockout prevents neuronal injury and behavioral impairment induced in a transgenic mouse model by a CXCR4-using HIV-1 glycoprotein 120. J. Immunol..

[bib39] Mocchetti I., Campbell L.A., Harry G.J., Avdoshina V. (2013). When human immunodeficiency virus meets chemokines and microglia: neuroprotection or neurodegeneration?. J. Neuroimmune Pharmacol..

[bib40] Morris S.R., Woods S.P., Deutsch R., Little S.J., Wagner G., Morgan E.E., Heaton R.K., Letendre S.L., Grant I., Smith D.M. (2013). Dual-mixed HIV-1 coreceptor tropism and HIV-associated neurocognitive deficits. J. Neurovirol..

[bib41] Ndhlovu L.C., Umaki T., Chew G.M., Chow D.C., Agsalda M., Kallianpur K.J., Paul R., Zhang G., Ho E., Hanks N. (2014). Treatment intensification with maraviroc (CCR5 antagonist) leads to declines in CD16-expressing monocytes in cART-suppressed chronic HIV-infected subjects and is associated with improvements in neurocognitive test performance: implications for HIV-associated neurocognitive disease (HAND). J. Neurovirol..

[bib42] Necula D., Riviere-Cazaux C., Shen Y., Zhou M. (2020). Insight into the roles of CCR5 in learning and memory in normal and disordered states. Brain Behav. Immun..

[bib43] Necula D., Riviere-Cazaux C., Shen Y., Zhou M. (2021). Insight into the roles of CCR5 in learning and memory in normal and disordered states. Brain Behav. Immun..

[bib44] Ojeda-Juárez D., Shah R., Fields J.A., Harahap-Carrillo I.S., Koury J., Maung R., Gelman B.B., Baaten B.J., Roberts A.J., Kaul M. (2020). Lipocalin-2 mediates HIV-1 induced neuronal injury and behavioral deficits by overriding CCR5-dependent protection. Brain Behav. Immun..

[bib45] Pulliam L., Sun B., Rempel H. (2004). Invasive chronic inflammatory monocyte phenotype in subjects with high HIV-1 viral load. J. Neuroimmunol..

[bib46] Raybuck J.D., Hargus N.J., Thayer S.A. (2017). A GluN2B-selective NMDAR antagonist reverses synapse loss and cognitive impairment produced by the HIV-1 protein tat. J. Neurosci..

[bib47] Robertson K.R., Smurzynski M., Parsons T.D., Wu K., Bosch R.J., Wu J., McArthur J.C., Collier A.C., Evans S.R., Ellis R.J. (2007). The prevalence and incidence of neurocognitive impairment in the HAART era. Aids.

[bib48] Ru W., Tang S.J. (2016). HIV-1 gp120Bal down-regulates phosphorylated NMDA receptor subunit 1 in cortical neurons via activation of glutamate and chemokine receptors. J. Neuroimmune Pharmacol..

[bib49] Ru W., Tang S.J. (2017). HIV-associated synaptic degeneration. Mol. Brain.

[bib50] Rush T., Martinez-Hernandez J., Dollmeyer M., Frandemiche M.L., Borel E., Boisseau S., Jacquier-Sarlin M., Buisson A. (2018). Synaptotoxicity in Alzheimer's disease involved a dysregulation of actin cytoskeleton dynamics through cofilin 1 phosphorylation. J. Neurosci..

[bib51] Sacktor N. (2018). Changing clinical phenotypes of HIV-associated neurocognitive disorders. J. Neurovirol..

[bib52] Sacktor N., Skolasky R.L., Seaberg E., Munro C., Becker J.T., Martin E., Ragin A., Levine A., Miller E. (2016). Prevalence of HIV-associated neurocognitive disorders in the multicenter AIDS cohort study. Neurology.

[bib53] Saylor D., Dickens A.M., Sacktor N., Haughey N., Slusher B., Pletnikov M., Mankowski J.L., Brown A., Volsky D.J., McArthur J.C. (2016). HIV-associated neurocognitive disorder--pathogenesis and prospects for treatment. Nat. Rev. Neurol..

[bib54] Shen W., Proost P., Li B., Gong W., Le Y., Sargeant R., Murphy P.M., Van Damme J., Wang J.M. (2000). Activation of the chemotactic peptide receptor FPRL1 in monocytes phosphorylates the chemokine receptor CCR5 and attenuates cell responses to selected chemokines. Biochem. Biophys. Res. Commun..

[bib55] Shepherd A.J., Loo L., Mohapatra D.P. (2013). Chemokine co-receptor CCR5/CXCR4-dependent modulation of Kv2.1 channel confers acute neuroprotection to HIV-1 glycoprotein gp120 exposure. PLoS One.

[bib56] Smith L.K., Babcock I.W., Minamide L.S., Shaw A.E., Bamburg J.R., Kuhn T.B. (2021). Direct interaction of HIV gp120 with neuronal CXCR4 and CCR5 receptors induces cofilin-actin rod pathology via a cellular prion protein- and NOX-dependent mechanism. PLoS One.

[bib57] Tang Q., Jiang J., Liu J. (2015). CCR5 blockade suppresses melanoma development through inhibition of IL-6-stat3 pathway via upregulation of SOCS3. Inflammation.

[bib58] Torres-Muñoz J.E., Van Waveren C., Keegan M.G., Bookman R.J., Petito C.K. (2004). Gene expression profiles in microdissected neurons from human hippocampal subregions. Brain Res. Mol. Brain Res..

[bib59] Tran P.B., Banisadr G., Ren D., Chenn A., Miller R.J. (2007). Chemokine receptor expression by neural progenitor cells in neurogenic regions of mouse brain. J. Comp. Neurol..

[bib60] Tyner J.W., Uchida O., Kajiwara N., Kim E.Y., Patel A.C., O'Sullivan M.P., Walter M.J., Schwendener R.A., Cook D.N., Danoff T.M. (2005). CCL5-CCR5 interaction provides antiapoptotic signals for macrophage survival during viral infection. NatMed.

[bib61] Wang Q., Yuan W., Yang X., Wang Y., Li Y., Qiao H. (2020). Role of cofilin in Alzheimer's disease. Front. Cell Dev. Biol..

[bib62] Wang Y., Liu M., Lu Q., Farrell M., Lappin J.M., Shi J., Lu L., Bao Y. (2020). Global prevalence and burden of HIV-associated neurocognitive disorder: a meta-analysis. Neurology.

[bib63] Westmoreland S.V., Alvarez X., deBakker C., Aye P., Wilson M.L., Williams K.C., Lackner A.A. (2002). Developmental expression patterns of CCR5 and CXCR4 in the rhesus macaque brain. J. Neuroimmunol..

[bib64] Winston A., Arenas-Pinto A., Stöhr W., Fisher M., Orkin C.M., Aderogba K., De Burgh-Thomas A., O'Farrell N., Lacey C.J., Leen C. (2013). Neurocognitive function in HIV infected patients on antiretroviral therapy. PLoS One.

[bib65] Wright E.J., Grund B., Cysique L.A., Robertson K.R., Brew B.J., Collins G., Shlay J.C., Winston A., Read T.R., Price R.W. (2015). Factors associated with neurocognitive test performance at baseline: a substudy of the INSIGHT Strategic Timing of AntiRetroviral Treatment (START) trial. HIV Med..

[bib66] Zheng J., Thylin M.R., Ghorpade A., Xiong H., Persidsky Y., Cotter R., Niemann D., Che M., Zeng Y.C., Gelbard H.A. (1999). Intracellular CXCR4 signaling, neuronal apoptosis and neuropathogenic mechanisms of HIV-1-associated dementia. J. Neuroimmunol..

[bib67] Zhou M., Greenhill S., Huang S., Silva T.K., Sano Y., Wu S., Cai Y., Nagaoka Y., Sehgal M., Cai D.J. (2016). CCR5 is a suppressor for cortical plasticity and hippocampal learning and memory. Elife.

[bib68] Zhou Y.J., Chen J.M., Sapkota K., Long J.Y., Liao Y.J., Jiang J.J., Liang B.Y., Wei J.B., Zhou Y. (2020). Pananx notoginseng saponins attenuate CCL2-induced cognitive deficits in rats via anti-inflammation and anti-apoptosis effects that involve suppressing over-activation of NMDA receptors. Biomed. Pharmacother..

